# Morphology-based deep learning approach for predicting adipogenic and osteogenic differentiation of human mesenchymal stem cells (hMSCs)

**DOI:** 10.3389/fcell.2023.1329840

**Published:** 2023-11-30

**Authors:** Maxwell Mai, Shuai Luo, Samantha Fasciano, Timilehin Esther Oluwole, Justin Ortiz, Yulei Pang, Shue Wang

**Affiliations:** ^1^ Department of Mathematics, Southern Connecticut State University, New Haven, CT, United States; ^2^ Department of Chemistry, Chemical and Biomedical Engineering, University of New Haven, West Haven, CT, United States; ^3^ Department of Cellular and Molecular Biology, University of New Haven, West Haven, CT, United States; ^4^ Department of Mechanical and Industrial Engineering, University of New Haven, West Haven, CT, United States

**Keywords:** HMSCs, osteogenic differentiation, adipogenic differentiation, transfer learning, deep learning, CNN, resnet

## Abstract

Human mesenchymal stem cells (hMSCs) are multipotent progenitor cells with the potential to differentiate into various cell types, including osteoblasts, chondrocytes, and adipocytes. These cells have been extensively employed in the field of cell-based therapies and regenerative medicine due to their inherent attributes of self-renewal and multipotency. Traditional approaches for assessing hMSCs differentiation capacity have relied heavily on labor-intensive techniques, such as RT-PCR, immunostaining, and Western blot, to identify specific biomarkers. However, these methods are not only time-consuming and economically demanding, but also require the fixation of cells, resulting in the loss of temporal data. Consequently, there is an emerging need for a more efficient and precise approach to predict hMSCs differentiation in live cells, particularly for osteogenic and adipogenic differentiation. In response to this need, we developed innovative approaches that combine live-cell imaging with cutting-edge deep learning techniques, specifically employing a convolutional neural network (CNN) to meticulously classify osteogenic and adipogenic differentiation. Specifically, four notable pre-trained CNN models, VGG 19, Inception V3, ResNet 18, and ResNet 50, were developed and tested for identifying adipogenic and osteogenic differentiated cells based on cell morphology changes. We rigorously evaluated the performance of these four models concerning binary and multi-class classification of differentiated cells at various time intervals, focusing on pivotal metrics such as accuracy, the area under the receiver operating characteristic curve (AUC), sensitivity, precision, and F1-score. Among these four different models, ResNet 50 has proven to be the most effective choice with the highest accuracy (0.9572 for binary, 0.9474 for multi-class) and AUC (0.9958 for binary, 0.9836 for multi-class) in both multi-class and binary classification tasks. Although VGG 19 matched the accuracy of ResNet 50 in both tasks, ResNet 50 consistently outperformed it in terms of AUC, underscoring its superior effectiveness in identifying differentiated cells. Overall, our study demonstrated the capability to use a CNN approach to predict stem cell fate based on morphology changes, which will potentially provide insights for the application of cell-based therapy and advance our understanding of regenerative medicine.

## Introduction

Mesenchymal stem cells (MSCs) have great potential for tissue engineering, regenerative medicine, and cell-based therapy due to their capacity for self-renewal and multipotency. Under certain chemical or biophysical stimulation, MSCs can be differentiated into various lineages, including osteoblasts, adipocytes, neurons, and chondrocytes (Augello and De Bari, 2010; Zhao et al., 2022a; Zhao et al., 2022b; Fasciano et al., 2023). MSCs can be isolated from various sources, including bone marrow, adipose tissue, placenta, umbilical cord or umbilical cord blood, respectively (Han et al., 2019). MSCs also possess various physiological effects, such as maintenance of tissue homeostasis, regeneration, and immunomodulatory properties, making them valuable for cell-based therapeutic applications (Zhou et al., 2021). MSCs offer considerable potential for regenerative medicine and therapeutic research; however, clinical trials utilizing MSCs face challenges such as variations in donor-derived cells, stability of stemness, differentiation capacity, and production inconsistency (Zhou et al., 2021). To meet the demand for a large number of functional stem cells for successful clinical translation, such as tissue regeneration, effective quality control of MSCs functions is required for high-quality, consistent, large-scale biomanufacturing of MSCs (Dwarshuis et al., 2017; Aijaz et al., 2018).

Although MSCs have been studied for decades, it is highly challenging to exclusively differentiate MSCs into a single desired cell type. Consequently, the identity and purity of the resulting cell population are critical for cell-based therapies. At present, the evaluation of the identity and purity of cell populations derived from MSCs typically involves measuring specific marker genes, or a combination of such markers. However, this method of classification raises concerns regarding the selection and specificity of these marker genes. Furthermore, current approaches for characterizing MSCs functions are lacking in clinical relevance, throughput, and robustness, highlighting the necessity for an automatic and robust method for quality control in MSCs functions.

Recently, it has been reported that MSCs functions, particularly differentiation potential, relate to cell morphology by exploiting advances in high-resolution microscopic imaging (Nombela-Arrieta et al., 2011; Singh et al., 2014; Kim et al., 2022a). For example, MSCs morphology has been correlated with differentiation capacity (Matsuoka et al., 2013; 2014; Lan et al., 2022a) and passage number (Lo Surdo and Bauer, 2012). Recent advancements in machine learning provide opportunities for predicting stem cell fate by utilizing large datasets of stem cell characteristics (Fan et al., 2017; Ashraf et al., 2021; Zhu et al., 2021). Among these machine learning methods, deep learning techniques have emerged as powerful tools to predict and identify stem cell patterns and lineage relationships (Kusumoto and Yuasa, 2019; Ren et al., 2021). These models can identify key features such as molecular signatures, cell morphology, and gene expression that influence stem cell fate, allowing for precise differentiation predictions. Deep learning algorithms can analyze this data to develop predictive models that accurately forecast the fate of stem cells, such as their differentiation into specific cell types, including osteocytes, adipocytes, or neurons. Machine learning algorithms have also been employed to predict MSC osteogenic potential (Matsuoka et al., 2013; Lan et al., 2022a), microenvironmental cues (Vega et al., 2012; Chen et al., 2016), and neural stem cell differentiation and blastocyst formation (Liao et al., 2021; Zhu et al., 2021). However, the majority of machine learning-based approaches rely on datasets collected from fixed cells rather than live cells. This method, exemplified by techniques like immunofluorescent staining, is time-consuming and uneconomical. Thus, there is an urgent need for an effective deep learning-based approach that can accurately predict and identify the fate of stem cells without the need for cell fixation and staining.

Recently, there has been growing interest in identifying differentiated stem cells based on accurate cellular morphology recognition using a simple microscope setup, thanks to the use of convolutional neural networks (CNNs) (Matsuoka et al., 2013; Dursun et al., 2021; Kim et al., 2022a; Chen et al., 2023). Matsuoka *et al.* has applied Ridge Regression as the machine learning modeling method to quantitatively predict cellular osteogenic potential (Matsuoka et al., 2013). Waisman *et al.* trained a CNN with transmitted light microscopy images to distinguish pluripotent stem cells from early differentiated cells (Waisman et al., 2019). Zhu et al. developed a deep learning-based platform to predict neuron stem cells (NSCs) differentiation using brightfield images without labelling (Zhu et al., 2021). Kusumoto et al. developed an automated deep learning-based system to identify endothelial cells derived from induced pluripotent stem cells (Kusumoto et al., 2018). Recently, Lan et al. developed a deep learning model called osteogenic convolutional neural network (OCNN) based on single-cell laser scanning confocal microscope (LSCM) images to predict osteogenic differentiation of rat bone marrow mesenchymal stem cells (rBMSCs) (Lan et al., 2022b). The OCNN model demonstrated its potential in predicting osteogenic drug effects, biomaterial development for bone tissue engineering, and cell-matrix interaction research. A transfer learning-based approach was utilized as the feature extractor predicting, with four well-performing models (VGG 19, InceptionV335, Xception, and DenseNet121) pre-trained on ImageNet. With over 85% accuracy, the results demonstrated the potential of a computer vision based method for identifying stem cell differentiation (Kim et al., 2022b). More recently, Zhou *et al.* introduced a predictive model for classifying hMSC differentiation lineages using the k-nearest neighbors (kNN) algorithm (Zhou et al., 2023). It provided accurate prediction of lineage fate on different types of biomaterials as early as the first week of hMSCs culture with an overall accuracy of 90.63% on the test data set. Although various CNN approaches have been employed to predict cell differentiation based on cellular morphology, achieving high prediction accuracy and precision remains a challenge. In response, our study leveraged innovative methodologies, integrating live-cell imaging with advanced deep learning techniques, specifically using a Convolutional Neural Network (CNN), to achieve exceptional prediction efficiency in identifying adipogenic and osteogenic differentiated hMSCs. Although several deep-learning based methods have been utilized to efficiently predict stem cell fate based on microscopic images, there is a still emerging need to identify and predict stem cell lineages based on live-cell imaging without fixation and staining. This motivated our work. Moreover, in order to choose appropriate deep-learning approaches, we have reviewed previous studies and most current deep-learning models. We systematically developed and evaluated four distinct CNN models: VGG 19, Inception V3, ResNet 18, and ResNet 50, to discern the cellular morphology changes associated with adipogenic and osteogenic differentiation. These four models were chosen based on their performance regarding accuracy, parameters, and performance in other deep-learning applications (Saber et al., 2021; Sahinbas and Catak, 2021; Palanivel and Nallasamy, 2023). Recently, ResNet 18 and ResNet 50 are most popular networks in classification of stem cell differentiation (Waisman et al., 2019; Chen et al., 2023; Kim et al., 2023). However, a comprehensive comparison of these two models with other models (VGG 19, Inception V3) has not been investigated, to the best of our knowledge. Our comprehensive analysis spanned multiple time points, ranging from 1 day to 15 days. We placed a primary focus on essential performance metrics such as accuracy, area under the Receiver Operating Characteristic curve (AUC), sensitivity, precision, and F1-score, applying these to both binary and multi-class classification of differentiated cells.

## Materials and methods

### Cell culture

Human Bone Marrow Derived Mesenchymal Stem Cells (hMSCs) were acquired from Lonza and PromoCell. According to the manufacturer, hMSCs were isolated from normal adult human bone marrow withdrawn from bilateral punctures of the posterior iliac crests of normal volunteers. Four vials of cells are purchased from different volunteers with different ages, which indicate the heterogeneity of hMSCs. hMSCs were cultured in mesenchymal stem cell basal medium MSCBM (PT-3238, Lonza) with GA-1000, L-glutamine, and mesenchymal cell growth factors (PT-4105, Lonza). Cells were cultured in 10 cm tissue culture dishes at 37°C and 5% CO_2_ in a humidified incubator. Cells were maintained regularly with medium change every 3 days and passaged using 0.25% EDTA-Trypsin (Invitrogen).

### hMSCs osteogenic differentiation

Osteogenic induction medium were prepared by adding Osteogenic Differentiation SingleQuotsTM Supplements (PT-4120), which include dexamethasone, L-glutamine, ascorbate, penicillin/streptomycin, MCGS, β-glycerophosphate into 170 mL of hMSC osteogenic differentiation basal medium (PT-3924, Lonza). To induce osteogenesis, hMSCs were plated at the concentration of 3 × 10^3^ per cm^2^ of tissue culture surface area in a 12- well plate. Cells were incubated at 37°C in a humidified atmosphere of 5% CO_2_ to allow cells to adhere. Following incubation, MSC basal medium was replaced with osteogenesis induction medium. A control group of hMSCs were cultured in basal MSC medium without osteogenic induction.

### hMSCs adipogenic differentiation

Adipogenic induction medium were prepared by adding Adipogenic Differentiation SingleQuots Supplements (PT-4135), which include h-insulin, L-glutamine, MCGS, dexamethasone, indomethacin, IBMX, and GA-1000, into 170 mL of adipogenic differentiation medium. To initiate adipogenesis, hMSCs were seeded onto tissue culture surfaces at a density of 3 × 10^3^ cells per square centimeter in a 12-well plate. After incubating for 24 h at 37°C in a humidified atmosphere containing 5% CO_2_ to promote cell adhesion, the MSC basal medium was substituted with an adipogenic induction medium. A control group of hMSCs was grown in MSC basal medium without the addition of induction factors.

### Alkaline phosphatase activity (ALP) staining

To quantify hMSCs osteogenic differentiation, cells were stained for alkaline phosphatase (ALP) using the alkaline phosphatase kit using a modified protocol. For live staining, hMSCs were stained using AP live stain at the concentration of 10x stock solution for 30 min according to the manufacturers’ instructions. For nucleus staining, Hoechst 33,342 staining solution was prepared in 1x PBS at 1:2000 dilution and added to cells for 15 min. The cells were then washed three times with 1 × PBS, 15 min each time, before taking images.

### Data acquisition and preprocessing

Images were captured using the ZOE Fluorescent Cell Imager with an integrated digital camera (BIO-RAD). All bright field images were taken after 1, 2, 3, 5, 7, 10, and 13 days of differentiation. Our data set contains 2,336 images taken at varying times after the initial culturing of the cells and divided into four groups: control, adipogenic, osteogenic, and adipogenic + osteogenic. The source images are gray scale with a resolution of 2,592 × 1,944 pixels.

The image preprocessing steps are as follows: 1) Resizing each image with bilinear interpolation and then converted to RGB format using Floyd-Steinberg dithering. 2) Normalizing RGB values by mean and standard deviation, with specific parameters detailed in Supplementary Table S1 RGB Normalization Values. 3) Cropping the images to match the input size required by the models. For the training data, this cropping is performed randomly, while for the testing data, a center crop is applied to ensure consistent results during testing. 4) Horizontal reflection was applied to increase the diversity and reduce the risk of overfitting.

The models utilized in our study were pretrained on the ImageNet1k dataset, a vast repository comprising over one million images categorized into one thousand distinct classes. Intriguingly, this dataset predominantly encompasses images of non-cellular subjects, with the majority of classes representing animals or household objects. Furthermore, the dataset exhibits fine-grained classification, exemplified by the presence of multiple distinct classes for closely related species, such as four different crab species and three distinct lobster species. Consequently, these pretrained models can be conceptualized as having undergone training not only in image classification but also in image differentiation. To adapt these models for our specific tasks, we introduced an additional densely connected layer featuring softmax activation, enabling them to produce probability distributions for each class. Additionally, all models were equipped with a stochastic gradient descent with momentum (SGDM) optimizer, characterized by a learning rate (α) set to 0.001 and momentum (β) set to 0.99, while employing categorical cross-entropy loss. Importantly, prior to training, we initialized each model’s weights based on their respective pretraining, with none of the convolutional layers being frozen. This decision was guided by the substantial dissimilarity between our dataset and the ImageNet1k dataset. Allowing all convolutional weights to be trainable permitted the models to leverage their pretraining “knowledge” as a foundational starting point, expediting the transfer of this knowledge into a completely novel domain.

### Transfer learning

Transfer learning is defined as applying a model trained on a general task to a new related task (You et al., 2019). Building a model using only cell images as training data is often not the most practical strategy since it requires large computational resources, and high quality labeled data is scarce. In addition, the deeper a network becomes (i.e., the more layers it has), the more training data it requires to converge on a best estimate for all parameters. Pre-trained convolutional neural networks (CNNs) have been trained on large-scale data sets and have learned general feature representations that capture meaningful patterns and structures in images of all types. In order to properly adapt these models to our task, we provide additional training data that is used to fine tune the parameters of the final layers in the network (Paszke et al., 2019). This fine-tuning process helps customize the model for our particular application while benefiting from the general knowledge the pretrained model has already learned.

### Evaluation metrics

To assess the performance of both binary and multi-class classification models, it necessitated the utilization of two distinct sets of evaluation metrics to evaluate their respective performances. For binary classification, we classified cells without differentiation as negative class, while cells exhibiting adipogenic differentiation were classified as positive class. In the context of multi-class classification, one-vs-rest (OvR) strategy was applied, where one class is treated as positive and the rest of the classes are combined into the negative class.

To evaluate and compare the different model performance, the true positive (*tp*), true negative (*tn*), false positive (*fp*), and false negative (*fn*) values were calculated. Then, five major measurements, including accuracy, precision, recall, F1 Score, and AUC, were calculated as follows, Eqs 1–4.

$$Accuracy=\frac{tp+tn}{tp+fp+fn+tn}$$ (1)

$$Precision=\frac{tp}{tp+fp}$$ (2)

$$Recall=\frac{tp}{tp+fn}$$ (3)

$$F1\;Score=\frac{2*Recall*Precision}{Recall+Precision}$$ (4)

Precision, also known as repeatability, quantifies the extent to which repeated measurements conducted under consistent conditions yield comparable outcomes (Eq. 2). In probabilistic terms, precision denotes the likelihood of a correct classification when the model predicts a positive label. On the other hand, recall, or sensitivity, is defined as the ratio of true positives to the sum of true positives and false negatives (Eq. 3). One can conceptualize recall as the proportion of correctly classified values, given that the true class for those values is positive. An important facet of precision and recall lies in their equilibrium relationship. Assuming that true positives and true negatives remain constant, elevating precision necessitates a corresponding reduction in recall. This adjustment occurs because the mitigation of false positives entails an increase in false negatives. Therefore, to quantify this trade-off, we calculate the harmonic mean of precision and recall, commonly known as the F1 score, Eq. 4. It is worth noting that precision, recall, and F1 score were compared exclusively for the binary classification because they are calculated assuming only two output classes.

The other two metrics, accuracy and area under ROC curve (AUC), were calculated and compared for both binary and multi-class classification. Accuracy is a fundamental metric used to assess classification models, representing the ratio of correct predictions to total number of predictions. AUC measures the probability that a random positive is positioned to a random negative example. AUC ranges in value from 0 to 1, with 0 indicating a model with completely incorrect predications and 1 indicting a model with entirely accurate predictions. AUC is desirable for the following two reasons: 1) AUC is scale-invariant. It measures how well predictions are ranked, rather than their absolute values. 2) AUC is classification-threshold-invariant. It measures the quality of the model’s predictions irrespective of what classification threshold is chosen. However, AUC is originally designed for binary classification. To apply AUC value for our multi-class classification, we utilized the “one vs. rest” method, i.e., we calculated binary AUC sores for each class independently and then averaged the four binary classification AUC scores as overall AUC score for multiclass. This is crucial, particularly considering our smallest class, “adipogenic + osteogenic,” which we anticipated would be challenging for the models to distinguish due to its multi-class nature.

## Results

### Datasets and procedures

**FIGURE 1:**
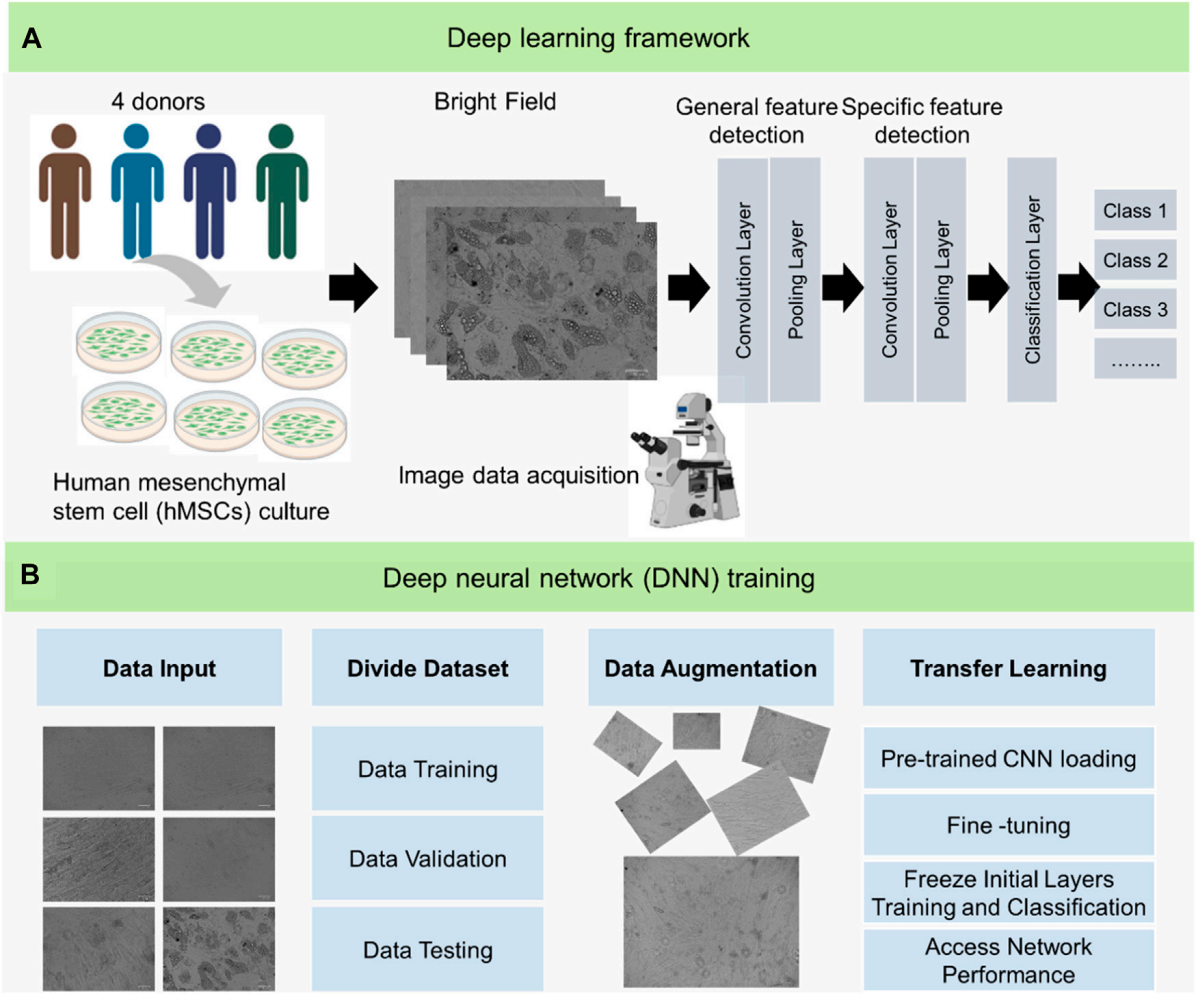
Schematic Illustration of the deep learning (DL) framework and deep neural network (DNN) training process used to identify mesenchymal stem cell differentiation. **(A)** Illustration of overall deep learning framework. Mesenchymal stem cells were acquired from four different donors. Bright field images of hMSCs with different treatments were obtained for classification. **(B)** Illustration of the process of deep neural network (DNN) training. The raw image data were initially obtained and divided into different datasets: training, validation, and testing sets on a ratio of 60:20:20. To increase the datasets, the images were cropped to increase the total number of datasets. Finally, the datasets were trained, tested, and validated using transfer learning.

Upon stimulation, hMSCs undergo a morphology change, transitioning from a spindle shape to a round shape. Consequently, we captured images on days 1, 3, 5, 7, 10, 13 and 15 of hMSCs undergoing adipogenic and osteogenic differentiation. For comparison, a positive control without induction was conducted. It is worth noting that a group of cells exposed to both adipogenic and osteogenic induction media was included to evaluate the CNN training model. In total, our dataset comprises 2,336 images, spanning control, adipogenic, osteogenic, and combined adipogenic + osteogenic groups. A schematic illustration of the deep learning framework and the deep neural network training process is depicted in Figure 1. After collecting the raw image data, general features like cell morphological changes are detected to form the convolution and pooling layers. Subsequently, specific features such as calcium deposition during osteogenic differentiation and lipid vacuole formation during adipogenic differentiation are identified. Finally, the dataset is classified into different groups based on these distinctive features, as illustrated in Figure 1A. To leverage the benefits of large neural networks while working with a limited dataset and preserving the predictive efficacy of our model, we pre-trained four different model architectures: VGG 19, Inception V3, ResNet 18, and ResNet 50 on the ImageNet1k dataset. This dataset is a vast repository containing over one million images categorized into one thousand distinct classes. For both binary and multi-class classification, all images were partitioned into three distinct sets, ensuring a balanced distribution. This resulted in a train-validation-test ratio of 3:1:1. It is important to note that this balanced partitioning ensured a roughly even distribution of each of the four classes across the training, validation, and test datasets. As a result, the training dataset for multi-class classification included 1,407 images, the validation set contained 473 images, and the test set had 456 images. For binary classification, the training set had 935 images, the validation set 313 images, and the testing set 304 images, as shown in Figure 1B.

**FIGURE 2:**
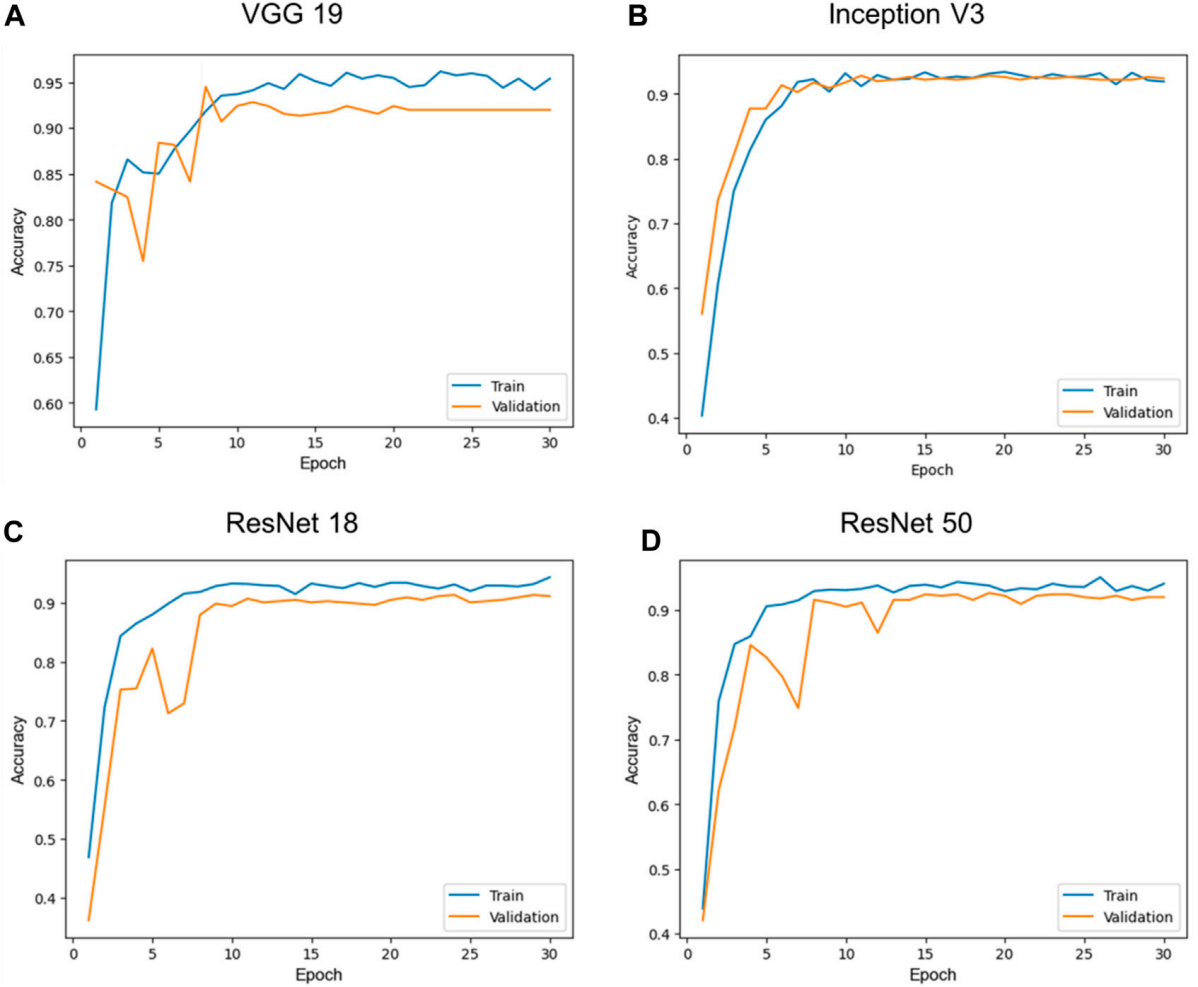
Comparison of training and validation accuracy per training epoch of different models, VGG 19 **(A)**, Inception V3 **(B)**, ResNet 18 **(C)**, and ResNet 50 **(D)**. All CNN networks achieved results close to 100% of accuracy after 15 training epochs.

Following each training epoch, the model was systematically evaluated with a single pass over the validation dataset, and the training data was shuffled. Subsequently, we selected the model with the highest validation accuracy as our final choice. Four pre-trained model architectures, VGG 19, Inception V3, ResNet 18, and ResNet 50 were subsequently compared to assess their network performance in terms of accuracy and area under the receiver operator characteristic curve (AUC). All models were trained and evaluated using the same data set splits for a total of 30 epochs before the final results were compared. Additionally, all convolutional layers were initialized based on the weights obtained during each model’s pretraining on the ImageNet1k dataset. Figures 2A–D showed the comparison of training and validation accuracy per training epoch of VGG 19, Inception V3, ResNet 18 and ResNet 50. All these four model architectures exhibited high validation accuracy (higher than 90%) after 15 training epochs. We observed that extending the training epoch count might not enhance the outcomes. Out of these four networks, Inception V3 stood out with closely aligned training and validation accuracy and required fewer than 10 epochs to achieve approximately 90% validation accuracy. Both ResNet 18 and ResNet 50 demonstrated comparable training and validation accuracy trends. Increasing the depth of network from 18 to 50 marginally enhanced the validation accuracy, Figures 2C, D. It is worth noting that VGG’s training and validation curves show a significant difference in accuracy, with the model achieving notably higher accuracy on the training dataset compared to the validation dataset. Furthermore, as the number of training epochs increases, the curves do not converge to the same value, a clear indicator of overfitting. To address this, implementing early stop becomes essential to achieve improved convergence and strike the balance between model complexity and generalization.

### Binary classification

**FIGURE 3:**
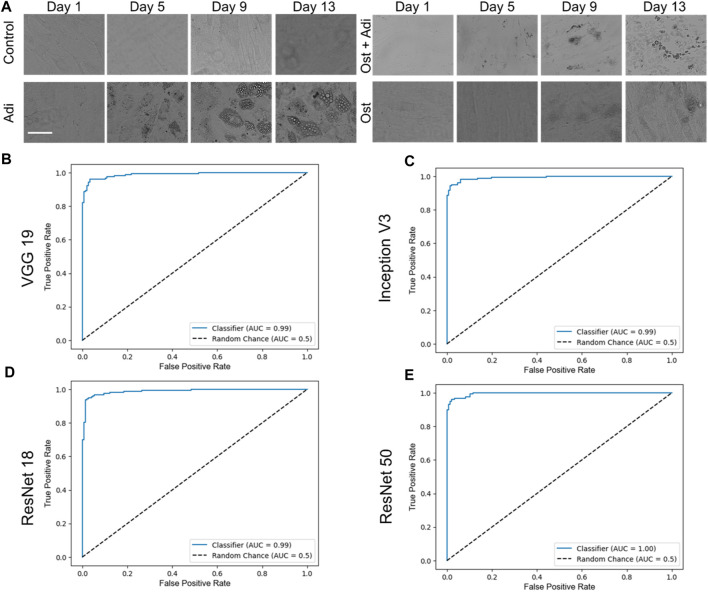
Binary classification and respective ROC curves of four different testing models, VGG 19, Inception V3, ResNet 18, and ResNet 50. **(A)** Brightfield images of hMSCs under varying conditions. Control: cells were cultured in basal medium without induction; Adi: cells were induced for adipogenesis; Ost: cells were induced for osteogenesis; Ost + Adi: cells were cultured in ostegenic and adipogenic induction medium with 1:1 ratio. Scale bar: 100 µm. ROC curves of VGG 19 **(B)**, Inception V3 **(C)**, Resnet 18 **(D)**, and ResNet 50 **(E)**.

To evaluate the performance of the four CNN networks, namely, VGG 19, Inception V3, ResNet 18, and ResNet 50, a binary classification was first conducted to identify adipogenic differentiated cells, as illustrated in Figure 3A. In our study, images characterized by a distinct adipogenic differentiation profile were designated as the positive class (Figure 3A, Adi group), while images without characteristics were categorized as members of the negative class (Figure 3A, control group). Figure 3B–D showed the ROC curves of these four different models, VGG 19, Inception V3, ResNet 18, and ResNet 50. For a comprehensive evaluation and comparison of these models for binary classification tasks, various performance metrics, including accuracy, AUC, precision, sensitivity, and F1—score, were assessed and analyzed at multiple time points (day 1, day 2, day 3, day 5, day 7, day 10, day 13, and day 15), as summarized in Table 1.

**TABLE 1:** The performance of each model for binary classification.

Model	Dataset	Accuracy	AUC	Sensitivity	Precision	F1 - score
VGG 19	Day 1	0.9211	0.9972	1.0000	0.8636	0.9268
	Day 2	0.9487	0.9974	1.0000	0.9091	0.9524
	Day 3	0.9750	0.9950	0.9500	1.0000	0.9744
	Day 5	0.9250	0.9850	0.9500	0.9048	0.9268
	Day 7	0.9750	0.9950	0.9500	1.0000	0.9744
	Day 10	1.0000	1.0000	1.0000	1.0000	1.0000
	Day 13	0.9333	1.0000	0.9000	1.0000	0.9744
	Day 15	0.9744	1.0000	0.9500	1.0000	0.9744
	**Overall**	0.9572	0.9895	0.9618	0.9557	0.9587
Inception V3	Day 1	0.8158	0.9806	0.9474	0.7500	0.8372
	Day 2	0.9487	1.0000	0.9000	1.0000	0.9474
	Day 3	0.9500	1.000	0.9000	1.0000	0.9474
	Day 5	0.9250	1.0000	0.9000	0.8696	0.9302
	Day 7	0.9750	0.9850	0.9500	1.0000	0.9744
	Day 10	1.0000	1.0000	1.0000	1.0000	1.0000
	Day 13	1.0000	1.0000	1.0000	1.0000	1.0000
	Day 15	1.0000	1.0000	1.0000	1.0000	1.0000
	**Overall**	0.9507	0.9926	0.9618	0.9438	0.9527
ResNet18	Day 1	0.9737	1.000	0.9474	1.0000	0.9730
	Day 2	0.9487	0.9921	0.9000	1.0000	0.9474
	Day 3	0.8000	0.9825	0.6000	1.0000	0.7499
	Day 5	0.9500	0.9750	1.000	0.9091	0.9524
	Day 7	0.9750	0.9750	0.9500	1.0000	0.9744
	Day 10	0.9474	1.0000	0.8889	1.0000	0.9412
	Day 13	0.9000	1.0000	0.8500	1.0000	0.9189
	Day 15	1.0000	1.0000	1.000	1.0000	1.0000
	**Overall**	0.9375	0.9890	0.8917	0.9859	0.9365
ResNet 50	Day 1	1.0000	1.0000	1.0000	1.0000	1.0000
	Day 2	0.9231	1.0000	0.8500	1.0000	0.9189
	Day 3	0.8000	1.0000	0.6500	1.0000	0.7500
	Day 5	0.9750	1.0000	1.0000	0.9524	0.9756
	Day 7	0.9750	0.9975	0.9500	1.0000	0.9744
	Day 10	1.0000	1.0000	1.0000	1.0000	1.0000
	Day 13	1.0000	1.0000	1.0000	1.0000	1.0000
	Day 15	1.0000	1.0000	1.0000	1.0000	1.0000
	**Overall**	0.9572	0.9958	0.9236	0.9932	0.9571

VGG 19 consistently exhibited high accuracy and AUC values across all evaluation days, with an overall accuracy and F1-score of 0.9572 and 0.9587, respectively. It demonstrated excellent sensitivity and precision, especially on day 10, where it achieved perfect scores. Inception V3 displayed a strong overall performance, with an overall accuracy of 0.9507 and F1-score of 0.9527. Although Inception V3 demonstrated a lower accuracy on day 1, it rapidly improved to achieve accuracy levels and matched VGG 19 from day 2 onwards, eventually reaching perfect accuracy (1.0000) on days 6, 7, and 10. ResNet 18, on the other hand, showed remarkable accuracy initially, but experienced some fluctuations, reaching a maximum of 1.0000 accuracy on day 7. Overall, ResNet 18 displayed fluctuating performance, resulting in an overall F1-score of 0.9365. While it achieved outstanding outcomes on day 1 and day 15, it experienced a decline in scores on day 3. Nonetheless, it consistently maintained high levels of precision and sensitivity. Finally, ResNet 50 consistently performed exceptional accuracy, maintaining a perfect score (1.0000) on multiple days, indicating robust and consistent performance. Its overall F1-score is 0.9571. It achieved perfect accuracy and AUC values on multiple days and demonstrated high sensitivity and precision, indicating robust and consistent binary classification capabilities.

In summary, all four models displayed strengths and weaknesses in various aspects of their performance. ResNet 50 and VGG 19 emerged as the top-performing models in terms of accuracy, with ResNet 50 achieving perfect accuracy on all days. Inception V3 also performed well, while ResNet 18 exhibited variable performance but still maintained robust precision and sensitivity. Therefore, the choice of the most suitable model may depend on specific task requirements and priorities among these performance metrics.

### Multi-class classification

**FIGURE 4:**
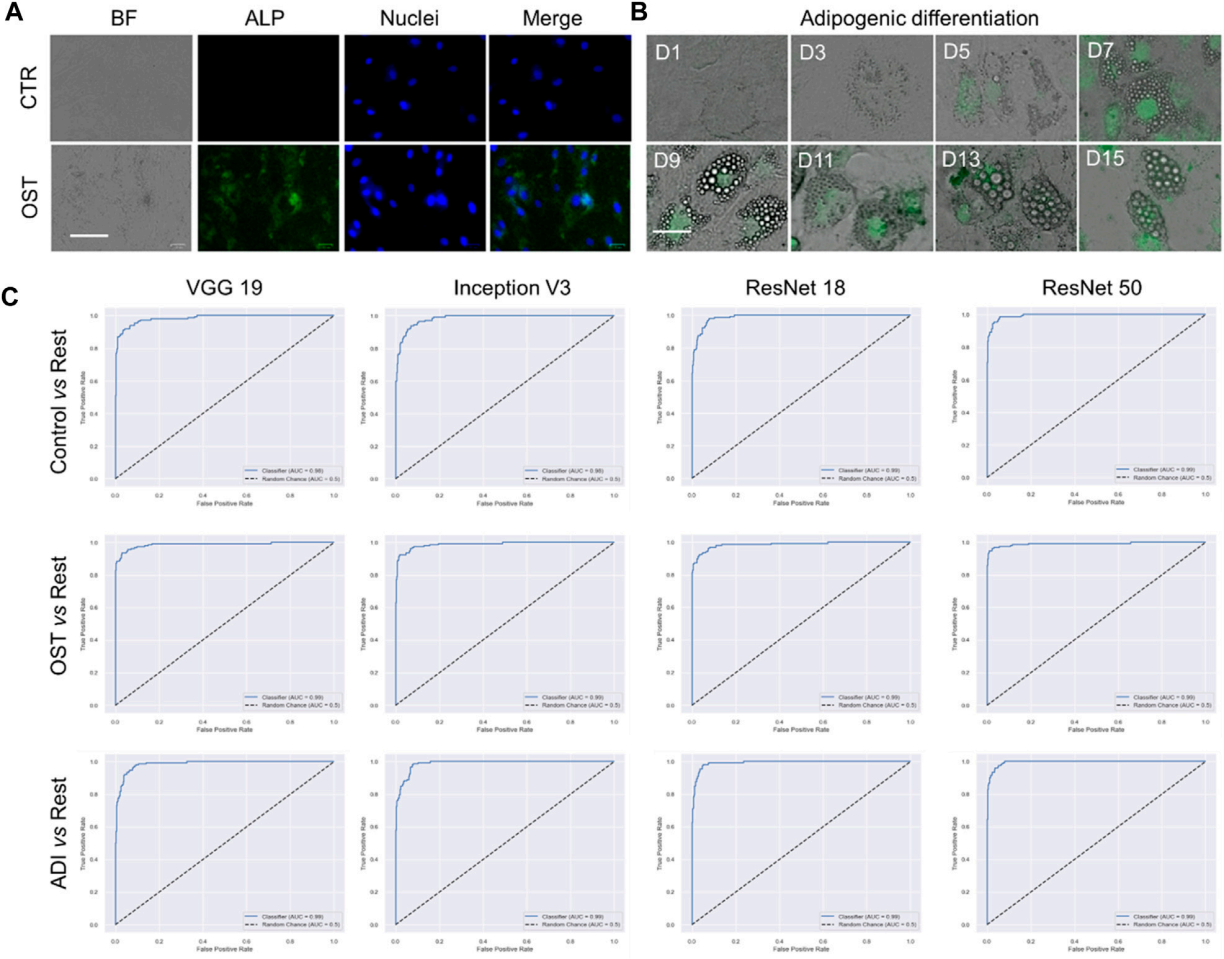
Multi-class classification of control, adipogenic differentiation, and osteogenic differentiation. **(A)** Representative images of hMSCs under different conditions. Control: cells were cultured in basal medium without induction; OST: cells were induced for osteogenesis. Scale bar: 100 µm. Green: ALP staining; Blue: cell nucleus stained with HoeChst 33,342. **(B)**. Brightfield images of hMSCs under adipogenic differentiation conditions at different time points. Green fluorescence indicates lipid marker (Bodipy). Scale bar: 50 µm. **(C)** ROC curves of VGG 19, Inception V3, Resnet 18, and ResNet 50.

We proceeded to conduct multi-class classification using all datasets: control, osteogenic differentiation, and adipogenic differentiation. As shown in Figure 4A, hMSCs under osteogenic and adipogenic differentiation exhibited distinct morphological changes compared to the control group. In the control group, where hMSCs were cultured in basal medium without differentiation induction, they maintained spindle shapes. In contrast, osteogenic-induced hMSCs transitioned from a spindle to a cuboidal shape as they differentiated and mineralized. Similarly, adipogenic-induced hMSCs transitioned from a spindle to a cuboidal shape and then formed lipid vacuoles, as depicted in Figure 4B. Observable morphological changes led us to hypothesize that the four pre-trained convolutional neural network models, namely, VGG 19, Inception V3, ResNet 18, and ResNet 50, could classify these three classes effectively. We then compared the performance of these models, focusing on accuracy and AUC, as shown in Table 2. Regarding accuracy, both VGG 19 and ResNet 50 displayed outstanding results with an overall score of 0.9474. Inception V3, though slightly behind with an accuracy of 0.9342, still demonstrated a strong performance. ResNet 18 closely matched the results of VGG 19, achieving an overall accuracy of 0.9408. When it came to AUC, ResNet 50 stood out with the highest overall value of 0.9936. VGG 19 and ResNet 18 also performed commendably with overall AUC scores of 0.9928 and 0.9925, respectively. Inception V3, with an overall AUC of 0.9899, showcased a competitive classification capability. In summary, all four models exhibited excellent performance, characterized by accuracy and AUC. ResNet 50 stood out with high accuracy and AUC, while VGG 19 also maintained high accuracy. Although Inception V3 and ResNet 18 had slightly lower accuracy and AUC values, their performance remained commendable.

**TABLE 2:** Comparison of accuracy and AUC of each model for multiclass classification.

Model	Dataset	Accuracy	AUC
VGG 19	Day 1	0.9825	1.0000
	Day 2	1.0000	1.0000
	Day 3	0.8814	0.9957
	Day 5	0.8833	0.9957
	Day 7	0.9661	0.9849
	Day 10	0.9649	1.0000
	Day 13	0.9184	1.0000
	Day 15	0.9821	1.0000
	Overall	0.9474	0.9928
Inception V3	Day 1	0.9474	0.9935
	Day 2	0.9831	0.9978
	Day 3	0.8305	0.9769
	Day 5	0.8333	0.9667
	Day 7	0.9322	0.9654
	Day 10	0.9649	1.0000
	Day 13	1.0000	1.0000
	Day 15	1.0000	1.0000
	Overall	0.9342	0.9899
ResNet18	Day 1	0.9649	0.9995
	Day 2	0.9661	0.9983
	Day 3	0.8305	0.9871
	Day 5	0.9500	0.9987
	Day 7	0.9492	0.9771
	Day 10	0.9649	1.0000
	Day 13	1.0000	1.0000
	Day 15	0.9107	0.9776
	Overall	0.9408	0.9925
ResNet 50	Day 1	0.9474	0.9991
	Day 2	0.9661	0.9987
	Day 3	0.8644	0.9833
	Day 5	0.9167	0.9867
	Day 7	0.9661	0.9893
	Day 10	0.9474	1.0000
	Day 13	1.0000	1.0000
	Day 15	0.9821	0.9990
	Overall	0.9474	0.9936

**FIGURE 5:**
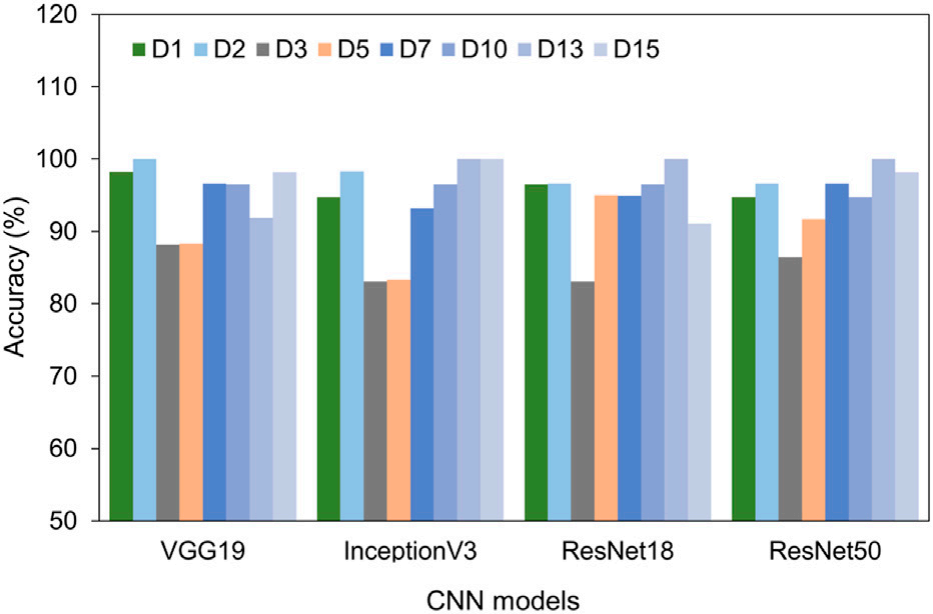
Comparison of test accuracy of different days using four different models, including VGG 19, Inception V3, ResNet 18, and ResNet 50.

Furthermore, we plotted and compared the accuracy of each model across different time points in Figure 5. All four models consistently achieved excellent performance with accuracy above 90% for the cells at day 1, 2, 7, 10, 13, and 15. Intriguingly, on days 3 and 5 of differentiation, the accuracy slightly dipped to around 88%. This decline might be due to the heterogeneity of the cells. Even with this minor reduction in accuracy on days 3 and 5, all models exhibited impressive overall performance, with VGG 19 and ResNet 50 being particularly noteworthy.

**FIGURE 6:**
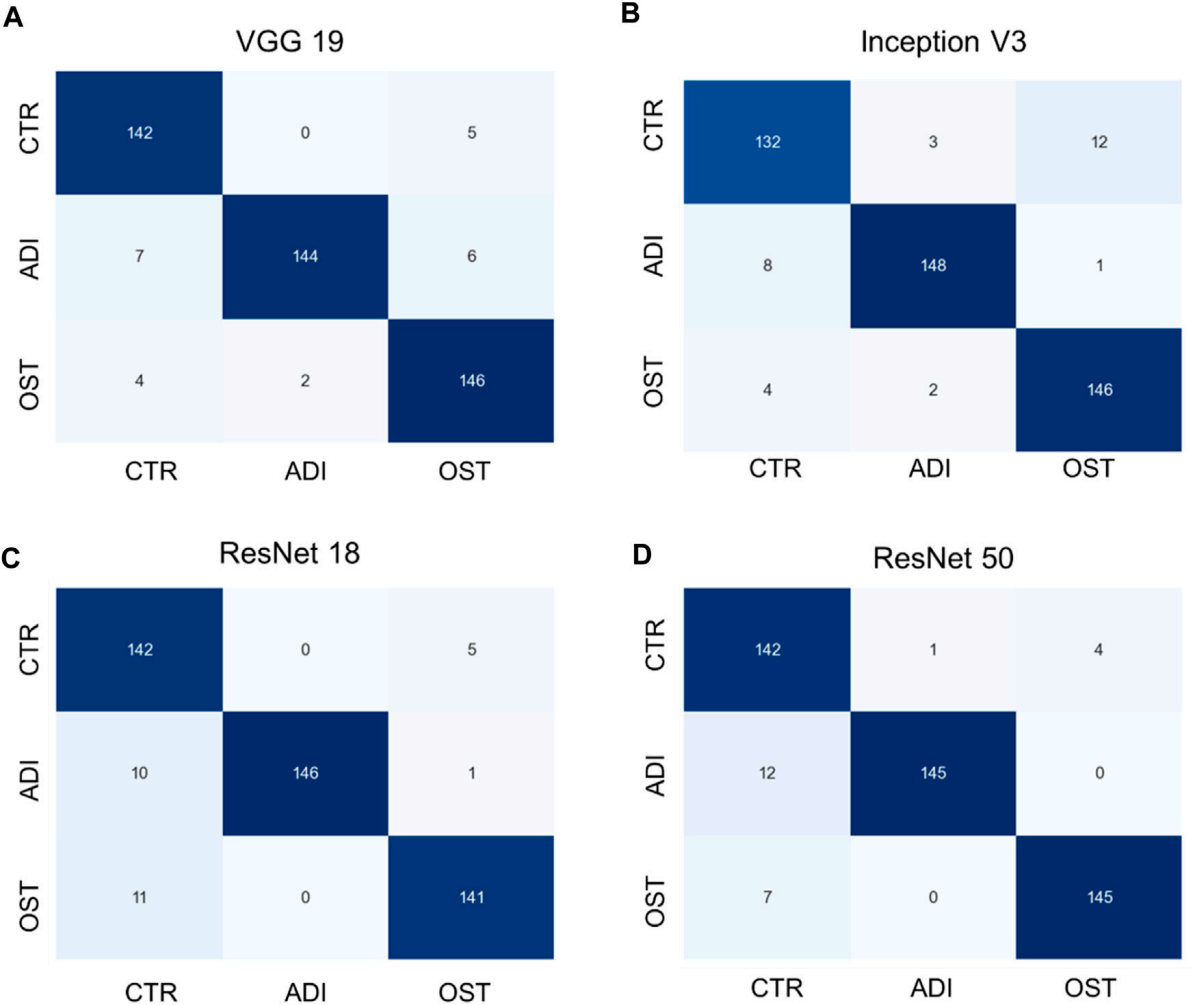
Confusion matrixes of multi-class classification of different models. **(A)** VGG 19 model, **(B)** Inception V3 model, **(C)** ResNet 18 model, and **(D)** ResNet 50 model.

**FIGURE 7:**
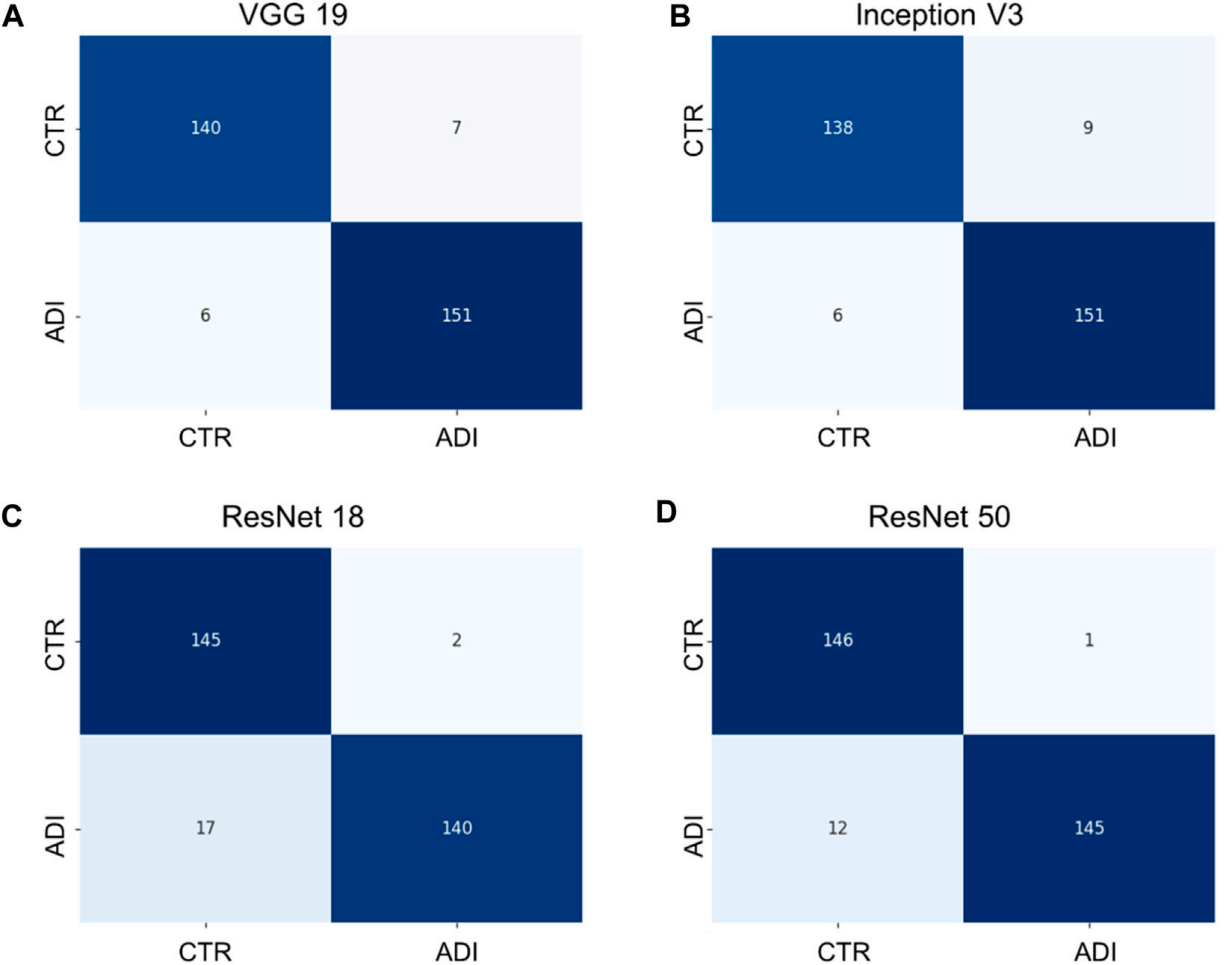
Confusion matrixes of binary classification of different models. **(A)** VGG 19 model, **(B)** Inception V3 model, **(C)** ResNet 18 model, and **(D)** ResNet 50 model.

Additionally, it is vital to recognize that each model has its unique set of parameters, as outlined in Supplementary Table S2. Although models with more parameters have the potential to manage more intricate scenarios, it is crucial to understand that continuously increasing the parameter count might not yield proportional benefits, especially when there’s limited training data. Moreover, larger models require more computational resources both for training and for producing results. Towards the conclusion, we generated confusion matrices represented in Figures 6, 7, providing a more comprehensive evaluation. Notably, in the multiclass task, Inception V3 did not perform as well as other models in distinguishing from the control group. In the binary classification task, however, the model demonstrated excellent precision but exhibited a trade-off with recall, particularly when compared to ResNet 50.

## Discussion

In this study, we developed and compared four convolutionary neural network models, including VGG 19, Inception V3, ResNet 18, and ResNet 50, to identify adipogenic and osteogenic differentiated cells based on morphology changes. To obtain better performance, all these four CNN models were pre-trained on the ImageNet1k dataset, a vast repository comprising over one million images categorized into one thousand distinct classes. Next, we evaluated the performance metrics of these four models in both binary and multi-class classification of differentiated cells across multiple time points (day 1, day 2, day 3, day 5, day 7, day 10, day 13, and day 15). The key performance metrics include accuracy, AUC, sensitivity, precision, and F1-score. Among all these four different models, ResNet 50 proves to be the most effective choice with its highest accuracy and AUC in both multi-class and binary classification tasks. Although VGG 19 matched ResNet 50’s accuracy in both tasks, ResNet 50 consistently outperformed with better AUC scores, emphasizing its effectiveness in identifying differentiated cells. As mentioned earlier, when comparing their performance, it is crucial to consider the parameters and resources of each network. Although ResNet 50 has more parameters, the substantial gain in accuracy in both binary and multi-class classification compensates for this limitation. In comparison, VGG 19 had slightly more parameters than ResNet 50. Thus, ResNet 50 excelled in accuracy and AUC while maintaining a moderate parameter count, making it the preferred choice for identifying adipogenic and osteogenic differentiated cells based on morphological changes. Furthermore, the marginally longer processing time of ResNet 50 was not a significant concern, especially when juxtaposed with traditional methods that take hours. It is also worth mention that the field of deep learning is dynamic, with continuous research leading to the development of new models and improvements. The models utilized in this study represent only a subset, and numerous other models and variations have emerged over the years. Four pre-trained models were selected for this study, all demonstrating satisfactory performance. This pioneering work aims to establish these models as benchmark models within the field. Moving forward, our focus will center on DenseNet and Vision Transformers (ViT). Recognizing the potential for ensemble methods to outperform individual models, we anticipate leveraging the strengths of all these architectures to enhance overall model performance.

These morphology-based CNN approaches offer significant advantages in predicting osteogenic and adipogenic differentiation, especially in the fields of biomanufacturing, cell-based therapy, and regenerative medicine. Moreover, these four models have the potential to predict other stem cell differentiation, such as, cell fate of human induced pluripotent stem cells (iPSCs), provided there are observable morphological changes associated with lineage adoption. These approaches offer automated tools for the precise discrimination between cell types, eliminating the need for manual feature classification, which is both time-consuming and expensive. Traditional approaches that involve staining biomarkers also rely heavily on specific staining reagents, markers, and cell types, which are factors that can affect prediction accuracy. In contrast, morphology-based CNN approaches are robust to variations in cell shape and adaptable to a wide range of experimental conditions. Their proficiency in handling vast datasets facilitates comprehensive analyses of cell differentiation processes, potentially hastening advancements in biomanufacturing, tissue engineering, and regenerative medicine. One of the key challenges in biomanufacturing lies in achieving a high purity of a specific lineage of stem cells, thus, one potential application in biomanufacturing is the integration of a deep-learning approach into automated, real-time analysis and feedback-controlled osteogenic differentiation. Specifically, we envision an automated platform capable of detecting changes in stem cell morphology, predicting stem cell fate, and controlling and directing osteogenic or adipogenic differentiation in real time. If an unexpected lineage is identified and reaches a certain percentage, this automated platform could adjust the microenvironment, for instance, by adding a chemical inducer to the bioreactor, to steer MSCs towards differentiating into a specific lineage.

Future efforts to improve the classification model should include incorporating more training data from a diverse range of donors and time points. Additionally, traditional staining assays will be performed to validate the model’s efficiency by staining for osteogenic biomarkers, such as ALP and Runx, as well as adipogenic markers like PPAR-γ. Subsequent models could also enhance their capabilities by incorporating more types of differentiated cells, including chondrogenic differentiation, and employing multi-label classifications without relying on independent classes. Such improvements would further enhance the versatility of these models, providing a deeper understanding of their precision in analyzing intricate aspects of cell morphology. An alternative strategy to identify a broader spectrum of cells involves the adoption of a Recursive Convolutional Neural Network (RCNN) architecture. Instead of classifying the entire image, an RCNN can systematically evaluate regions within each image, allowing for precise localization and identification of differentiated cells. This approach not only facilitates the independent identification of multiple cell types but also provides information about the spatial distribution and size of each cell cluster, enabling the computation of differentiation degrees. It offers a more streamlined and efficient alternative compared to using two separate models. Nonetheless, it is important to note that this technique requires more complex training data, necessitating the delineation and labeling of each cell, which poses a significant data annotation challenge. Despite these challenges, the RCNN approach holds substantial promise and has the potential to simplify the overall cell identification process.

## Conclusion

In this study, we developed and compared four convolutional neural network (CNN) models: VGG 19, Inception V3, ResNet 18, and ResNet 50, for the purpose of identifying adipogenic and osteogenic differentiated cells based on cellular morphological changes. We conducted a comprehensive evaluation of these models in both binary and multi-class classification of differentiated cells at various time points (day 1, day 2, day 3, day 5, day 7, day 10, day 13, and day 15), focusing on the key performance metrics that include accuracy, AUC, sensitivity, precision, and F1-score. Among these four models, both VGG 19 and ResNet 50 showed excellent performance with high accuracy for both binary (0.9572) and multi-class classification (0.9474). ResNet 50 showed consistent performance with high AUC (0.9936) for multi-class classification. Importantly, all these four models exhibited exceptional performance with the overall accuracy of more than 0.93, and overall AUC score of more than 0.94. By analyzing the daily images of differentiated cells, all these models can accurately detect subtle morphological changes within 1 day of differentiation. In summary, our study underscores the immense potential of using a CNN approach to predict stem cell fate based on cellular morphological changes of differentiated cells. This approach holds promise for enhancing the application of cell-based therapy and expanding our knowledge of regenerative medicine. Additionally, this non-invasive method, relying solely on basic bright-field microscope images, has the potential to facilitate biomanufacturing and the translation of these advancements into practical cell-based therapies.

## Data Availability

The original contributions presented in the study are included in the article/Supplementary Material, further inquiries can be directed to the corresponding authors.
