# Supplementary material for: Morphology-based deep learning approach for predicting adipogenic and osteogenic differentiation of human mesenchymal stem cells (hMSCs)
Source: Front Cell Dev Biol. 2023 Nov 30;11:1329840. doi: 10.3389/fcell.2023.1329840 (PMC10720363; doi:10.3389/fcell.2023.1329840)
Supplement: Supplementary file 1 [file DataSheet1.docx]

**Morphology-based Deep Learning Approach for Predicting Adipogenic and Osteogenic Differentiation of Human Mesenchymal Stem Cells (hMSCs)**

Maxwell Mai^1,ǂ^, Shuai Luo^2, ǂ^ , Samantha Fasciano^3^, Timilehin Esther Oluwole ^2^, Justin Ortiz^4^, Yulei Pang^1,*^, Shue Wang ^2,*^

^1^Department of Mathematics, Southern Connecticut State University, New Haven, CT, 06515, USA.

^2^Department of Chemistry, Chemical and Biomedical Engineering, University of New Haven, West Haven, CT, 06516, USA.

^3^Department of Cellular and Molecular Biology, University of New Haven, West Haven, CT, 06516, USA.

^4^Department of Mechanical and Industrial Engineering, University of New Haven, West Haven, CT, 06516, USA.

^ǂ^ M. M. and S. L. contributed equally.

*Corresponding Author:

Dr. Yulei Pang ([pangy1@southernc.edu](mailto:pangy1@southernc.edu)) is with Southern Connecticut State University, New Haven, CT, 06515, USA.

Dr. Shue Wang ([swang@newhaven.edu](mailto:swang@newhaven.edu)) is with the University of New Haven, West Haven, CT, 06516, USA.

**Supplemental Materials**

**Table S1**. Mean and standard deviations used for normalization groups by color channel

**Table S2**. Parameter counts for each of the models under consideration

**Table S1**. Mean and standard deviations used for normalization groups by color channel

| **Color Channel** | **R** | **G** | **B** |
| --- | --- | --- | --- |
| Mean | 0.485 | 0.456 | 0.406 |
| StdDev | 0.229 | 0.224 | 0.225 |

**Table S2**. Parameter counts for each of the models under consideration

| **Model** | **Parameter Count** |
| --- | --- |
| VGG 19 | 143.6 M |
| Inception V3 | 24 M |
| ResNet 18 | 11.7 M |
| ResNet 50 | 25.6 M |
